# The Fourth Konstantin Ivanov Intercontinental Magnetic Resonance Conference on Methods and Applications ICONS-4

**DOI:** 10.1007/s00723-022-01468-w

**Published:** 2022-03-24

**Authors:** G. Buntkowsky, D. Abergel, P. K. Madhu

**Affiliations:** 1 Institute of Inorganic and Physical Chemistry, Technical University of Darmstadt, 64287 Darmstadt, Germany; 2 Laboratoire des Biomolécules, Département de chimie, École Normale Supérieure, PSL, University, CNRS, Sorbonne Université, 75005 Paris, France; 3 TIFR Centre for Interdisciplinary Sciences, Tata Institute of Fundamental Research Hyderabad, 36/P Gopanpally Village, Ranga Reddy District, Hyderabad 500107 India

**ICONS** Organized during February 09–11, 2022, was the fourth edition of the on-line magnetic resonance conference series called Konstantin Ivanov **I**nter**CON**tinental Magnetic Resonance **S**eminar, named after our untimely deceased colleague and friend. The ICONS conferences are an off-shoot of the weekly Intercontinental NMR Seminar Series that started on April 8, 2020. This seminar series has enabled the communication and dissemination of research ideas among the magnetic research community in the times of the COVID-19 pandemic and will continue to do so beyond. In the framework of the ICONS series, until now, more than 130 scientists from five different continents have presented their recent results. While the weekly seminar series gives both early-stage and experienced researchers an opportunity to give seminar talks and interact with colleagues from all over the world, the ICONS conference is a platform for experienced researchers. The ICONS-4 conference attracted registrations from nearly 200 people from 30 countries (in the spirit of the meeting, covering six continents, Europe, North America, South America, Africa, Australia, and Asia) and spanned 17 time zones from Japan over Europe to the West Coast. The meeting talks were broadcast across the Zoom and YouTube platforms. The average combined attendance was around 100.

The ICONS seminar series is open to all areas of magnetic resonance and covers the full range of Magnetic Resonance, i.e., EPR, NMR, MRI, and their various hybrids. While the summer ICONS conferences (see reports in APMR [1, 3] for details) are equally broad in scope as the seminars, the spring conferences are focused on a narrower subject, such as techniques, where the interaction of electron and nuclear spins play a pivotal role (ICONS-2, see APMR [2] for details) or the various flavors of hyperpolarization and NMR signal enhancement (present ICONS-4 installment). The main goal of the ICONS-4 focus was to present an overview about the current state-of-the-art of Dynamic Nuclear Polarization (DNP), Parahydrogen-based techniques (PHIP, SABRE), Chemically Induced Nuclear Polarization (CIDNP), Nitrogen Vacancies (NV), and Optical Pumping (SEOP) with the idea to further support and stimulate interactions among groups employing these techniques in EPR, NMR and MRI. To achieve this goal, twelve speakers were selected among the leading experts in these fields and invited to report at the conference.

**Jan Henrik Ardenkjær-Larsen** Denmark, reported on the current frontier in clinical applications of dissolution-DNP in MRI. He discussed the recent development and applications of a highly automated, robust, and efficient DNP polarizers suitable for medical environments and in vivo applications to patients.

**Pierre-Jean Nacher** France, gave an overview about the techniques and applications of hyperpolarized noble gases (^129^Xe, ^3^He). He demonstrated that optical pumping creates near unity polarization in these gases and presented applications of this hyperpolarization in magnetometry, high-energy and neutron physics, and biomedical imaging.

**Eleonora Cavallari** Italy, gave a fascinating overview of new developments in the application potential of Parahydrogen-based hyperpolarized techniques in medical imaging and discussed the potential of current metabolites, and the challenges of making hyperpolarization process biocompatible.

**Thomas Meersmann** UK, discussed the application potential of hyperpolarized noble gases (^83^ K, ^129^Xe) in MRI applications in health care and the monitoring of gas distributions in hierarchically organized porous solids, such as, e.g., diesel catalysts.

**Eriks Kupče, Bruker** UK, introduced the concept of NMR supersequences for small molecule applications, leading to a substantial decrease in experimental time and significant increase in the sensitivity of NMR measurements. Combinations of several “normal” NMR experiments were shown that can be tailored for specific applications, for instance, the analysis and characterization of molecular structure of complex organic molecules.

**Lucio Frydman** Israel, discussed sensitivity-enhancement of the NMR spectra of bioliquids and solid-state NMR via chemical exchange saturation transfer (CEST). Employing CEST with the solvent the signals of imino, amino, amide, and hydroxy peaks in the 2D NOESY/TOCSY NMR spectra of nucleic acids, proteins, and saccharides can be increased by factors ranging from two- to tenfold. Finally, he showed that such techniques are also adaptable to solid-state NMR, e.g., by transferring polarization of surrounding media in wide-line NMR of ^14^ N, ^17^O, and ^33^S.

**Philip Kuchel** Australia, reported the application of dissolution DNP to study ^133^Cs^+^ in suspensions of human erythrocytes. Employing this technique, he investigated the transmembrane exchange in these cells and estimated the corresponding flux rates by statistical modelling.

**Silvia Cavagnero** USA, introduced the state-of-the-art low-concentration photo-CIDNP as an effective tool for the generation of hyperpolarization by employing tryptophane (Trp) as a model amino acid and proteins containing Trp residues in solution NMR. She discussed the creation of the photo-states via LEDs, the synthesis and application of selectively ^13^C labelled Trp as reporter-residue, and experiments in highly complex media, which are models for cellular media.

**Christian Degen** Switzerland, reported on recent progress in their efforts to utilize NV centers in diamonds as sensors for detecting single nuclear spins in their vicinity. After giving an overview about the detection of ^13^C spins of the diamond in the vicinity of the NV-center, he discussed the potential and challenges of detecting nuclear spins outside of the diamond.

**Michal Leskes** Israel, gave a fascinating overview about the application potential of endogenous paramagnetic metal ions in inorganic solids as hyperpolarization source in applications of DNP in materials science and surface NMR. She also discussed how the combination of endogenous and exogenous (e.g., biradicals) polarization sources can be forged into a powerful spectroscopic tool for structural characterizations of thin coatings and buried solid interphases.

**Simon Duckett** UK, discussed the application of biomarkers hyperpolarized via PHIP (or SABRE) as a cost-efficient alternative diagnostic tool to conventional positron emission tomography (PET). He introduced suitable reactive precursors as building blocks for hyperpolarized reporter molecules and demonstrated the detection of previously unseen reaction intermediates.

**Gaël de Paëpe** France, gave a report on the current state-of-the-art and technological developments in MAS-DNP. First, he discussed DNP-enabled natural abundance ^13^C-^13^C and ^15^ N-^13^C correlation experiments as tools for crystal structure determination of small molecules, protein aggregates, and ligand arrangement and proximities at the surface of nanocrystals. Then he introduced novel improved polarizing agents. In the last part of his presentation, he gave a stunning overview of their laboratory built sustainable cryogenic helium MAS for high-field DNP setup.

**Organization and Future Developments** The conference was organized by Daniel Abergel (ENS Paris, France), Gerd Buntkowsky (TU, Darmstadt, Germany), and P. K. Madhu (TIFR Hyderabad, India). Suman Saurav and Sreenidhi, TIFR Hyderabad, provided technical assistance. The conference and seminar series were sponsored by Alexander von Humboldt Foundation, Wiley, Springer, HyperSpin, and Adani. Following the scheme of a general MR conference in summer alternating with a specialized conference on cutting-edge topics in winter, there are already plans for a general ICONS5 in summer of 2022. For updates and the schedule of upcoming talks see the home page of the meeting ICONS-Seminar.
